# Trends in the risk of myocardial infarction among HIV-1-infected individuals relative to the general population in France: Impact of gender and immune status

**DOI:** 10.1371/journal.pone.0210253

**Published:** 2019-01-16

**Authors:** Aliou Baldé, Sylvie Lang, Aline Wagner, Jean Ferrières, Michèle Montaye, Pierre Tattevin, Laurent Cotte, Elisabeth Aslangul, Frédéric Bidégain, Antoine Chéret, Murielle Mary-Krause, Jean-Luc Meynard, Jean-Michel Molina, Marialuisa Partisani, Pierre-Marie Roger, Franck Boccara, Dominique Costagliola

**Affiliations:** 1 Sorbonne Université, INSERM, Institut Pierre Louis d’épidémiologie et de Santé Publique, IPLESP, Paris, France; 2 Laboratoire d'épidémiologie et de santé publique, Université de Strasbourg, Strasbourg, France; 3 Centre hospitalier universitaire de Rangueil, service de cardiologie B, Université de Toulouse, Toulouse, France; 4 INSERM, Institut Pasteur de Lille, Univ. Lille, CHU Lille, France; 5 Hôpital Universitaire Pontchaillou, service des maladies infectieuses et USI, Rennes, France; 6 INSERM U1052, Hospices Civils de Lyon, Hôpital de la Croix Rousse, service des maladies infectieuses et tropicales, Lyon, France; 7 Sorbonne Paris Cité, Université Paris Descartes, APHP, Hôtel Dieu, service de médecine interne, Paris, France; 8 APHP, Hôpital Avicenne, service de maladie infectieuse, Bobigny, France; 9 Sorbonne Paris Cité, Université Paris Descartes, Paris, Centre hospitalier de Tourcoing, service des maladies infectieuses, Tourcoing, France; 10 APHP, Hôpital Saint-Antoine, service des maladies infectieuses et tropicales, Paris, France; 11 Sorbonne Paris Cité, Université de Paris Diderot Paris, INSERM, UMRS 941, APHP, Hôpital Saint-Louis, service des maladies infectieuses et tropicales, Paris, France; 12 Hôpitaux Universitaires de Strasbourg, Le Trait D’Union, centre de soins de l’infection par le VIH, Strasbourg, France; 13 Université de Nice Sophia-Antipolis, Centre hospitalier universitaire de Nice, groupe hospitalier l’Archet, service d’infectiologie, Nice, France; 14 Sorbonne Université, INSERM, APHP, Hôpital Saint-Antoine, service de cardiologie, Paris, France; Rush University, UNITED STATES

## Abstract

We examined trends in the MI incidence and age at MI diagnosis among adults living with HIV-1 between 2000 and 2009, by comparison with the French MI registries, by gender. Age standardized incidence rates and standardized incidence-ratios (SIRs) were estimated for individuals included in the French hospital database on HIV (n = 71 204, MI = 663) during three periods: 2000–2002, 2003–2005 and 2006–2009. Median ages at MI diagnosis were compared using the Brown-Mood test. Over the study periods, the absolute rate difference and relative risks were higher in women than in men in 2000–2002 and 2006–2009, with respective SIRs 1.99 (1.39–2.75) and 1.12 (0.99–1.27) in 2006–2009. The trends were different for men and women with a decreasing trend in SIRs in men and no change in women. In both sexes, among individuals with CD4 ≥500/μL and controlled viral-load on cART, the risk was no longer elevated. Age at MI diagnosis was significantly younger than in the general population, especially among women (-6.2 years, p<0.001; men: -2.1 years, p = 0.02). In HIV-1-positive adults, absolute rate difference and relative risks and trends of MI were different between men and women and there was no additional risk among individuals on effective cART.

## Introduction

Previous studies have shown a higher risk of myocardial infarction (MI) among people living with HIV (PLHIV) than in the general population [1–4]. HIV-infected individuals have a higher prevalence of traditional cardiovascular risk factors (CvRFs) than the general population [3, 5] but this does not fully explain the elevated risk of cardiovascular disease [4, 6]. Of note, the relative risk of MI was higher in HIV-infected women than in men in the only two studies reporting analyses of the risk by gender [1, 3]. Several studies have also shown an increased risk of MI among HIV-infected individuals exposed to first-generation protease inhibitors (PIs) such as indinavir, amprenavir, fos-amprenavir and lopinavir [7–9]. Independently of cardiovascular risk factors and antiretroviral therapy, HIV replication and a low CD4 cell nadir have been associated with an increased risk of MI [4, 10]. Recently, analyses of the Kaiser Permanente database showed that the risk of MI was lower when the current CD4 cell count was ≥500/μL than when it was <500/μL [6], and that the risk of MI among individuals living with HIV was no longer elevated in 2011 [11]. In Denmark, Rasmussen et al. observed a decline in the relative risk of MI among HIV-infected relative to HIV-uninfected individuals from 2006, while the risk was relatively stable or increasing from 1995 to 2005 [12]. In recent studies [11–12] results were not reported separately for men and women.

Age-related morbidities were found to occur much earlier in individuals infected with HIV than in uninfected individuals, when the difference in age distribution between the two populations was not taken into account [13]. Althoff et al [14], studying age at MI diagnosis in a 97%-male population, found no difference with the general population after adjusting for the age distribution.

The aim of the present study was to examine trends in the incidence of MI among individuals living with HIV enrolled in the French Hospital Database on HIV (FHDH-ANRS CO4), by comparison with the general population using data from the French coronary heart disease (CHD) registries, during the period 2000–2009. Our main focus was on first whether the relative risk, the absolute rate difference and the trends differ by gender, second whether the risk remain elevated in individuals living with HIV with CD4 cell recovery and third, whether MI was diagnosed at a younger age in PLHIV, separately for each gender.

## Methods

### Study population

MI incidence rates in the general population were obtained from the French CHD registries located in northern (Lille), north-eastern (Strasbourg) and south-western France (Toulouse), each covering about one million inhabitants [15].

Created in 1989, the French Hospital Database on HIV (FHDH) is a nationwide, open, prospective cohort of adults living with HIV managed in 70 hospitals. The project was approved by the French data protection authority (CNIL) on 27 November 1991 (Journal Officiel, 17 January 1992). Data submitted by the participating hospitals to the data center are anonymized on the basis of the individual’s last name, first name, and day and month of birth, then encrypted. Therefore, data available in the database are considered to be indirectly identifying. Because of the need to return to the individuals’ medical records to validate a diagnosis or to collect additional data for specific research projects, the CNIL approval for a correspondence list kept in the hospital was obtained in 1999. The only cohort inclusion criteria are HIV-1 or HIV-2 infection and written informed consent. The database included data on 120 542 individuals living with HIV seen at least once between 1 January 1992 and 31 December 2009, with a median follow-up of 5.6 years [interquartile range (IQR) = 2.1–11.3]. In 2009, individuals enrolled in the FHDH represented 53% of all individuals qualifying for free healthcare in France because of HIV/AIDS [16].

This analysis focused on HIV-1-infected individuals followed in the FHDH between 1 January 2000 and 31 December 2009 and aged between 35 and 74 years at any time during the study period. Individuals were excluded if they only had inclusion visit and no recorded date of death, that is if they had no follow-up. Individuals with no available CD4 cell count or no viral load values in 2000–2009 were also excluded. Individuals from French overseas territories were not eligible because data are not available for the general populations in these territories. Individuals with a history of MI at enrolment were excluded from the analysis. All new MIs diagnosed between 2000 and 2009 were included in the analysis. The study period, corresponding to 10 years of follow-up, was divided into the early (2000–2002), intermediate (2003–2005) and late cART eras (2006–2009).

### Definition of myocardial infarction

In the FHDH, myocardial infarction is coded using the International Classification of Diseases 10th revision (ICD-10: codes I21 to I21.9) [17]. Cases were validated by a cardiologist using the American College of Cardiology/European Society of Cardiology definition of myocardial infarction [18]. In the French CHD registries, we used categories 1 (acute myocardial infarction) and 2 (coronary death) up to 2005. From 2006 onwards, the definition also included the newly introduced category 5 (acute coronary syndrome).

### Statistical methods

For each calendar period, individuals were included in the analysis if they had at least one visit during the relevant period. The follow-up start date was the date of inclusion in the FHDH or the start date of the calendar period, whichever occurred later. Therefore, one individual can participate to more than one period. In each period, individuals are censored in case of MI, lost to follow-up, death or at the end of the period which ever occur first. Crude incidence rates (S1 Table) for the age group and gender were estimated by dividing the number of cases by the number of person-years at risk. We calculated standardized incidence rates (IRs) and their 95% confidence intervals (CIs) by gender in each calendar period for the HIV-infected and general populations by weighting crude incidence rates by the age (5-year increments) structure of the individuals living with HIV followed in the FHDH between 2000 and 2009 [19]. In men and women from HIV-infected and general populations, standardized incidence rates in the early cART era (2000–2002) and the late cART era (2006–2009) were compared to the one in the intermediate cART era (2003–2005) by a t-test. Absolute rate difference and their 95% CIs were estimated by gender in each calendar period using the difference in IRs between the HIV-infected and general populations.

The standardized incidence ratios (SIRs) were used to compare the observed number of cases of MI among HIV-infected individuals with the expected number in the general population by gender in each calendar period. Confidence intervals for the SIRs were calculated with an exact method based on the Poisson distribution [20]. To study trends overtime for each gender, SIRs in the early cART era and in the late cART era were compared to the one in the intermediate cART era, using data stratified by age group, each stratum including information on the number of MIs and the corresponding time at risk. P values were derived from Poisson regression models for these comparisons. Also, P values of SIRs comparison between men and women were obtained by Poisson regression in each calendar period. SIRs were also estimated for cART-treated individuals whose CD4 cell count had been at least 500/μL continuously for at least 2 years and whose viral load was controlled (≤500 copies/mL) at the last assessment before MI diagnosis or the last follow-up visit in comparison to the general population separately for each gender. Follow-up started 2 years after the first CD4 cell count above 500/μL. At the start of follow-up, HIV-infected individuals had to have spent at least 6 months on cART, as calculated from the date of cART initiation. Follow-up was censored when the first CD4 cell count below 500/μL was recorded.

Age at MI diagnosis was estimated by gender and compared between the HIV-infected and general populations after adjusting for the differences in the age distribution, by using the indirect standardization method described by Shiels et al [13]. For each gender, expected median age at MI diagnosis in the general population was estimated and compared, using the Brown-Mood test [21], with the median age at MI diagnosis among HIV-infected individuals between 2000 and 2009.

## Results

As shown in S1 Fig, between 2000 and 2009, 71 204 individuals living with HIV were eligible, representing 403 903.2 person-years of follow-up (mean 5.7 years). A total of 663 incident MIs were validated, with 596 incident cases among 51 295 HIV-infected men and 67 incident cases among 19 909 HIV-infected women (S1 Fig). The MI incident cases distribution was 147 in 2000–2002, 221 in 2003–2005 and 295 in 2006–2009 (Table 1). The characteristics of the HIV-infected individuals in the three study periods are shown in Table 1. Men accounted for about three-quarters of the study population, and about one-half of the men were “men who have sex with men”. The proportion of intravenous drugs users (IVDU) fell across the study periods, and the median age rose from 39 to 43 years. The proportion of HIV-infected individuals with HIV RNA ≤500 copies/mL rose from 46% in the early cART period (2000–2002) to 64% in the late cART period (2006–2009). The proportion of individuals with CD4 cell counts ≥500/μL rose from 32% in the early cART period to 40% in the late cART period. The proportion of individuals with CD8 cell counts <1000/μL rose from 33% in the early cART period to 47% in the late cART period.

**Table 1:** Individual characteristics at entry in each calendar period^a^.

Characteristic	Early cART era (2000–2002)	Intermediate cART era (2003–2005)	Late cART era (2006–2009)
Number of HIV-infected individuals with at least 1 FHDH visit	43 628 (100)	51 007 (100)	58 866 (100)
New MI cases			
Men	132	205	259
Women	15	16	36
Gender and HIV transmission group			
MSM	16 037 (36.7)	18 595 (36.5)	21 443 (36.4)
IVDU men	6 974 (16.0)	6 635 (13.0)	5 993 (10.2)
IVDU women	2 515 (5.8)	2 531 (5.0)	2 350 (4.0)
Other men	9 828 (22.5)	12 111 (23.7)	14 526 (24.7)
Other women	8 274 (19.0)	11 135 (21.8)	14 554 (24.7)
Origin			
Sub-Saharan men	1 787 (4.1)	2532 (5.0)	3 480 (5.9)
Sub-Saharan women	1 661 (3.8)	2 784 (5.5)	4 379 (7.4)
Non sub-Saharan men	31052 (71.2)	34809 (68.2)	38 482 (65.4)
Non sub-Saharan women	9 128 (20.9)	10 882 (21.3)	12 525 (21.3)
Age	39 (36–46)	41 (37–47)	43 (38–49)
HIV RNA copies/mL^b^	819 (50–20 025)	148 (50–16 300)	50 (50–6 580)
HIV RNA ≤500 copies /mL	20 400 (46.8)	27 962 (54.8)	37 765 (64.1)
CD4 cells/μL^c^	377 (220–563)	411 (250–601)	433 (279–621)
CD4 cell counts ≥500 cells/μL	14 199 (32.5)	18 936 (37.1)	23 494 (39.9)
CD8 cells/μL^d^	887 (616–1 243)	908 (634–1 265)	872 (616–1 215)
CD8 cell counts <1000 cells/μL	14 332 (32.9)	18 886 (37.0)	27 693 (47.0)
CD4/CD8 ratio	0.4 (0.2–0.6)	0.4 (0.3–0.7)	0.5 (0.3–0.8)
CD4/CD8 ratio ≥1	2 161 (8.9)	3 451 (10.5)	5 731 (12.6)
CD4 nadir cells/μL	198 (80–341)	198 (81–335)	204 (90–335)
CD4 nadir cell counts <200 cells/μL	21 898 (50.2)	25 629 (50.2)	28 603 (48.6)
ARV treatment			
Naive	7 494 (17.2)	10 290 (20.2)	12 952 (22.0)
Previous ARV, no current ARV	2 828 (6.5)	4 751 (9.3)	7 876 (13.4)
Non cART ARV	4 162 (9.5)	1 935 (3.8)	1 049 (1.8)
cART^e^	29 144 (66.8)	34 031 (66.7)	36 989 (62.8)
AIDS	8 953 (20.5)	10 983 (21.5)	12 745 (21.7)
HBs antigen-positive	1 591 (3.6)	2 577 (5.1)	3 472 (5.9)
HCV antibody-positive	5 543 (12.7)	6 750 (13.2)	7 671 (13.0)
Smokers			
Unknown			30 656 (52.1)
Never			10 723 (18.2)
Previous			4 612 (7.8)
Current			12875 (21.9)
Person-years of follow-up			
Men	77 081.4	91 868.4	128 048.2
Women	24 015.2	32 373.3	50 516.7

Data are counts (proportions) and medians (interquartile range).

Abbreviations: MI, myocardial infarction, ARV, antiretroviral drugs; cART, combination antiretroviral therapy; HIV, human immunodeficiency virus; MSM, men who have sex with men, IVDU, intravenous drug users.

^a^ A given individual could be followed in more than one period.

^b^ HIV RNA data were available for 43 686 individuals (98.7%) in the early cART period, 51046 individuals (99.1%) in the intermediate cART period, and 58 866 individuals (100.0%) in the late cART period.

^c^ CD4 cell counts were available for 43 883 individuals (99.1%) in the early cART period, 51 153 individuals (99.3%) in the intermediate cART period, and 58 866 individuals (100.0%) in the late cART period.

^d^ CD8 cell counts were available for 24 193 individuals (55.5%) in the early cART period, 32 814 individuals (64.4%) in the intermediate cART period, and 45 419 individuals (77.2%) in the late cART period.

^e^ cART is defined as boosted protease inhibitor (PI) monotherapy, whatever the PI; dual therapy with 2 boosted PIs or 1 boosted PI plus 1 non-nucleoside reverse transcriptase inhibitor; treatment with an integrase inhibitor and/or anti-CCR5 drug; or a combination of ≥3 drugs.

### Trends in the incidence of MI, and comparison with the general population

**Fig 1:**
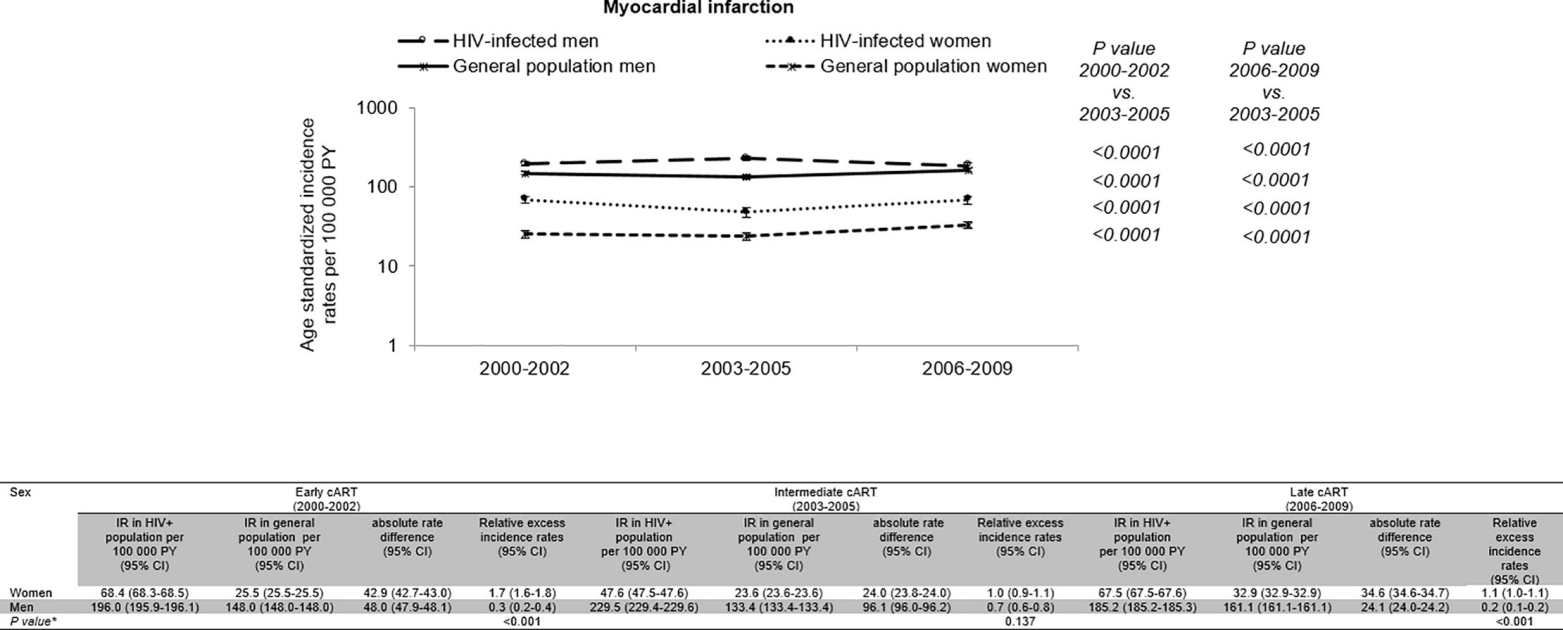
Myocardial infarction standardized incidence rates, absolute rate difference and corresponding 95% confidence intervals in the HIV-infected and general populations in calendar period. Standardization is based on the age and gender distribution of the HIV-infected population between 2000 and 2009. **Abbreviations**: cART, combination antiretroviral therapy; HIV, human immunodeficiency virus; CI, confidence interval; PY, person-years; IR, incidence rate. The absolute rate difference (shown with the 95% confidence interval) per 100 000 person-years was estimated as the difference in the age standardized incidence rate between HIV–infected individuals and the general population. For each gender, the relative excess incidence rate in each calendar period was obtained dividing the absolute rate difference by the incidence rate in the general population. * *P values* for the relative excess incidence rates were obtained by Poisson regression by comparison between men and women in each calendar period.

Differences in the age and gender distribution between the HIV-infected and general population are shown in S2 Fig. The HIV-infected population was younger than the general population. Age -standardized incidence rates of MI in the HIV-infected population (men and women) and general population (men and women) are shown in Fig 1 for each calendar period. The standardized incidence rates of MI changed significantly across the 3 calendar periods among HIV-infected men, HIV-infected women, or among men and women of the general population, remaining higher in the HIV-infected population than in the general population. The absolute rate difference of MI between PLHIV and the general population (shown in Fig 1 with the 95% CI) changed also across the calendar periods.

In HIV-infected men, there was a peak of age standardized incidence rate in 2003–2005 with age standardized incidence rates significantly higher than in 2000–2002 (p<0.0001) and in 2006–2009 (p<0.0001). The absolute rate difference of MI among men living with HIV relative to the male general population increased between 2000–2002 and 2003–2005 and then, decreased and was significantly smaller in 2006–2009 than in the previous two calendar periods. The absolute rate difference among men living with HIV in 2006–2009 was +24 MIs per 100 000 person-years in comparison to the male general population.

In HIV-infected women, however, the age standardized incidence rate was the lowest in 2003–2005. The absolute rate difference of MI among women living with HIV relative to the female general population fell significantly between 2000–2002 and 2003–2005, but increased between 2003–2005 and 2006–2009, remaining lower, however, than in 2000–2002. In 2006–2009, there were 35 additional MIs per 100 000 person-years among women living with HIV than in the female general population.

Of note the relative excess incidence rate in each calendar period obtained dividing the absolute rate difference by the incidence rate in the general population (that is equivalent to a relative risk change), was 170% in 2000–2002, 100% in 2003–2005 and 110% in 2006–2009 among women, 30% in 2000–2002, 70% in 2003–2005 and 20% in 2006–2009 among men and was higher in women than in men (p<0.001) except in 2003–2005 (p = 0.137).

### Risk of MI among HIV-infected individuals versus the general population

The SIRs for MI, calculated according to gender in each calendar period, are shown in Table 2. All the SIRs were significant, with the exception of the SIR among men living with HIV in the last calendar period. No significant difference in SIRs across the calendar periods was observed among women (p>0.05), for whom the estimated SIR in the last period was 1.99 (95% CI: 1.39–2.75). In contrast, the SIRs among men showed a significant increase from early to intermediate cART era (p = 0.028), and a significant decrease from intermediate to late cART era (p<0.001). The risk was only marginally elevated in the last calendar period, with a SIR of 1.12 (95% CI: 0.99–1.27). Comparing SIR among men and women living with HIV in each calendar period, the relative risk of MI was higher among women than men living with HIV in 2000–2002 (p = 0.008) and in 2006–2009 (p = 0.001) but not in 2003–2005 (p = 0.377).

**Table 2:** Standardized incidence ratios by gender in each calendar period.

Gender	Early cART era (2000–2002)		Intermediate cART era (2003–2005)		Late cART era (2006–2009)		*P* value 2000–2002 vs. 2003–2005	*P* value 2006–2009 vs. 2003–2005
	O/E	SIR (95% CI)	O/E	SIR (95% CI)	O/E	SIR (95% CI)		
Women	15/5.33	2.81 (1.58–4.64)	16/7.32	2.18 (1.25–3.55)	36/18.10	1.99 (1.39–2.75)	0.485	0.752
Men	132/97.10	1.35 (1.14–1.61)	205/117.96	1.74 (1.51–1.99)	259/230.80	1.12 (0.99–1.27)	0.028	<0.001
P value*		0.008		0.377		0.001		

Abbreviations: CI, confidence interval; O/E, observed/expected cases; SIR, standardized incidence ratio.

*P* values for the SIRs were obtained by Poisson regression by comparison two by two and their 95% CIs were estimated with an exact method based on the Poisson distribution.

*P values for the SIRs were obtained by Poisson regression by comparison between men and women in each calendar period.

Among HIV-infected individuals with CD4 cell counts ≥500/μL for at least 2 years and viral load ≤500 copies/mL on cART started at least 6 months previously, the risk was not higher than in the general population, with SIRs of 1.04 (95% CI: 0.12–3.76) for women and 0.99 (95% CI: 0.66–1.42) for men.

### Age at MI diagnosis

The median ages at MI diagnosis were respectively 48.7 and 44.0 years among men and women living with HIV. After adjustment for age differences, age at MI diagnosis remained significantly younger among both men and women living with HIV (p<0.05). By comparison with the general population, women living with HIV were diagnosed with MI at a much younger age than women from the general population, while the difference was much smaller for men, with respective median differences of -6.2 years and -2.1 years (Table 3).

**Table 3:** Age at diagnosis of myocardial infarction (MI) among HIV-infected individuals and the general population in France between 2000 and 2009.

MI	Observed age, HIV-infected population	Observed age, general population	Observed difference (years)	Expected age, general population	Real difference (years)^a^	P value^b^
Women	44.0 (40.5–51.7)	67.5 (57.5–72.5)	-23.5	50.2 (43.7–60.6)	-6.2	<0.001
Men	48.7 (43.4–55.3)	62.5 (52.5–67.5)	-13.8	50.8 (45.0–58.1)	-2.1	0.02

Age at MI diagnosis is shown as the median (interquartile range).

^a^ Real difference estimated as the difference between observed age at MI diagnosis in the HIV-infected population and expected age at MI diagnosis in the general population.

^b^ Brown-Mood test.

## Discussion

Relative to the general population, the risk of myocardial infarction among HIV-infected men in France peaked in 2003–2005 and fell during the last study period (2006–2009) to a marginally elevated relative risk of 1.12 (95% CI 0.99–1.27). In HIV-infected women, no such trend was found, the relative risk was higher than in men during 2000–2002 and 2006–2009 and not in 2003–2005 and it was 1.99 (95% CI 1.39–2.75) in 2006–2009. Interestingly, the relative risk was no longer elevated among either men or women with immune recovery (CD4 ≥500/μL) for at least 2 years and controlled viral load on cART. Age at MI diagnosis was significantly younger among HIV-infected individuals than in the general population, especially among women (-6.2 years, versus -2.1 years among men).

The main strengths of this study are the large number of HIV-infected individuals included and the large number of incident cases of myocardial infarction recorded and validated by gender in each calendar period. This allowed us to estimate IRs and SIRs by gender separately for each calendar period. However, the number of incident cases of MI among women in each calendar period (15 in 2000–2002, 16 in 2003–2005 and 36 in 2006–2009) yield a lack of precision in estimation of confidence intervals. Also, we were not able to assess the role of hepatitis B or C co-infection or from sub-Saharan origin as the number of MI cases were even smaller in these subgroups. In hepatitis C co-infected individuals, incident cases of MI were 1, 4 and 10 among women and 14, 30 and 40 among men in 2000–2002, 2003–2005 and 2006–2009 respectively. Given the insufficient number of MI cases in women in each calendar period, we did analyses only in the subgroup of men co-infected with hepatitis C or not. Relative to the male general population, the risk of MI in male co-infected with hepatitis C were significant and did not decrease across calendar period with a SIR of 2.04 (95% CI: 1.46–2.78) in 2006–2009. The relative risk of MI and trend across calendar period in men non co-infected with hepatitis C were similar to those previously found in HIV-infected men with a SIR of 1.04 (95% CI: 0.90–1.18) in 2006–2009. The relative risk of MI in men co-infected was significantly higher compared to that in non co-infected with hepatitis C in 2003–2005 (p = 0.001) and in 2006–2009 (p<0.001) (S2 Table). Hepatitis C co-infection may play a role in the observed trends of the risk of MI in HIV-infected individuals but we were unable to confirm it in women. In general, the SIR reasonably approximates the relative risk [22], and this holds true for MI, as its incidence in the general population is large enough not to be influenced by the relatively small fraction of HIV-infected individuals. Our control population was the general population. As elegantly shown in the COBRA study [23] the choice of the control group is particularly important and comparison with the general population may tend to overestimate the impact HIV infection over life styles factors such as smoking, illicit drugs use and others. Underreporting of MI in the FHDH database might be an issue, but cardiovascular risk has been a concern for physicians caring for HIV-infected individuals since the late 1990s, and also a topic of specific FHDH research. In addition, we found an elevated risk of MI in all three study periods and in both sexes, a result that could not be explained by underreporting. The definition of MI in the general-population registries changed in 2006 with the addition of category 5 (acute coronary syndrome), but there was a shift of some MI previously reported in category 1 (acute MI) to MI reported in category 5 and this change of definition is therefore unlikely to explain our results. As data collection on MI in the general-population registries and in FHDH started prior to the publication of the universal definition of MI in 2007 [18], we do not have data enabling us to distinguish between type 1 and type 2 MI.

In men living with HIV, we found a peak of absolute rate difference in 2003–2005 which decreased in 2006–2009 and a declining relative risk in men in the same period. In women living with HIV, the absolute rate difference in 2003–2005 was the lowest but relative risk did not changed over the three calendar periods. Klein et al in the USA observed a decreasing trend in the relative risk of MI from 1.3 (1.0–1.6) in 2004–2007 to a no elevated risk of 1.0 (0.7–1.4) in 2010–2011 in a 91% male HIV-positive and HIV-negative populations [11]. Interestingly, these latter authors found a relative risk of 1.3 (95% CI: 0.9–1.7) for the period 2008–2009. Rasmussen et al [12] in Denmark from the 76% men HIV-infected and uninfected cohorts, found that the relative risk of MI remained relatively stable from 1995–1999 to 2006–2008 and a significant decline over recent calendar periods (2009–2011 and 2012–2014). As neither teams reported their results separately for men and women, we cannot compare our results to theirs for the absence of trends in the relative risks in women but as more than two third (2/3) of their study populations was male the observed overall trend was probably dominated by the trend in men. The fact that our analysis stopped in 2009 may explain why we still observed a marginally elevated risk in the period 2006–2009 in men (1.12, 95% CI: 0.99–1.27).

What factors can explain the observed trends? We and others showed an elevated risk of MI associated with indinavir, lopinavir and amprenavir/fos-amprenavir use [8–9], while use of atazanavir has not been found associated with an increased risk of MI [24–25] and recently data on darunavir from the Data collection on Adverse Events of Anti-HIV Drugs (D:A:D) study group showed that the cumulative use of darunavir was independently associated with a small, but gradually increasing risk of cardiovascular disease of 59% per 5 years exposure [26]. As a matter of facts, trends in the use of these PI peaked in 2003–2005 in men in the FHDH (S3 Fig). In women the trends were different, with still increasing use of these PI in the last study period. A second factor that can be related to the observed trends is the level of CD8 in the study population. A CD8 cell count level above 1000/μL has been shown to be associated with an increased risk of MI [10, 27] and as shown in Table 1, the proportion of individuals with CD8 below this level increased overtime. Change in the proportion of smokers may also have contributed to the observed trends, however as smoking status has only been recorded in the database from 2005, we cannot verify this assumption.

The absence of an increased risk of MI observed here among individuals with immune recovery and controlled viral load on cART with SIRs of 1.04 (95% CI: 0.12–3.76) for women and 0.99 (95% CI: 0.66–1.42) for men is in line with results from Silverberg et al [6], who reported a non-significant relative risk of 1.18 (95% CI: 0.96–1.45) among individuals in the Kaiser Permanente database with a current CD4 cell count of at least 500/μL. This slightly higher risk than that found here (1.0) is likely explained by a difference in the definition of the studied population: a CD4 cell count ≥500/μL for 2 years and controlled viral load in our study, compared to only ≥500/μL in the most recent available CD4 cell count [6], as we previously showed both viral load and having had a low CD4 are associated with the risk of MI [10].

We found that the difference in age at MI diagnosis relative to the general population was larger among women (-6.2 years) than men (-2.1 years). Relative to uninfected individuals, Althoff et al [14] found no noteworthy difference in mean age at MI diagnosis (-0.11 years) in the 97%-male Veteran Aging Cohort of HIV-infected individuals. We have no clear explanation for this difference, but it should be noted that our MI population was larger (596 cases in men and 67 women) than that of Althoff with 291 MI. The higher relative risk of MI among women, and their younger age at diagnosis, could be due at least in part to the observation that traditional cardiovascular risk factors are more frequent in HIV-infected women than in both HIV-infected men and the female general population [5].

In conclusion, this study shows that in France the absolute rate difference and the relative risk of myocardial infarction among HIV-infected men declined in 2006–2009 but no such changes was observed in women over time. Interestingly, the absolute rate difference, the relative risk and age at MI diagnosis were gender-dependent. The relative risk in women living with HIV compared to the female general population was higher than in men except in 2003–2005. The relative risk is no longer elevated among individuals with immune recovery and controlled viral load on cART whether men or women. This study found a decreasing trend in the risk of MI which may be due to earlier treatment initiation with less toxic drugs and the resulting improvement in immunologic status. This decline was clearer for men than women; therefore, future studies should clarify why this gender difference exists.

## Supporting information

S1 Table Crude incidence rate for each calendar, period and global incidence rate. **Abbreviations**: CI, confidence interval; O, observed cases; PY, person-years; IR, incidence rate.
(DOCX)

S2 Table Standardized incidence ratios by gender in each calendar period among HCV antibody-positive men. **Abbreviations**: HCV, hepatitis C virus; CI, confidence interval; O/E, observed/expected cases; SIR, standardized incidence ratio; HCV Ab, hepatitis C antibody. *P* values for the SIRs were obtained by Poisson regression by comparison two by two and their 95% CIs were estimated with an exact method based on the Poisson distribution. *P values for the SIRs were obtained by Poisson regression by comparison between men and women in each calendar period.
(DOCX)

S1 Fig Flowchart of inclusions. **Abbreviations:** FHDH, French Hospital Database on HIV; VL, viral load; MI, myocardial infarction; PY, person-years.
(TIF)

S2 Fig Age and gender distribution of the HIV-infected and general populations. (TIF)

S3 Fig Trends in the use of protease inhibitors (PI) in men (A) and women (B) in FHDH-ANRS CO4. **Abbreviations:** ATV, atazanavir; DRV, darunavir; LPV, lopinavir; IDV, indinavir; APV, amprenavir; FPV, fos-amprenavir.
(TIF)
