# Supplementary material for: Trends in the risk of myocardial infarction among HIV-1-infected individuals relative to the general population in France: Impact of gender and immune status
Source: PLoS One. 2019 Jan 16;14(1):e0210253. doi: 10.1371/journal.pone.0210253 (PMC6334967; doi:10.1371/journal.pone.0210253)
Supplement: S1 Table — Abbreviations: CI, confidence interval; O, observed cases; PY, person-years; IR, incidence rate. (DOCX) [file pone.0210253.s001.docx]

**S1 Table**.

| Gender | Early cART era  (2000-2002) | | | Intermediate cART era  (2003-2005) | | | Late cART era  (2006-2009) | | | Global cART  (2000-2009) | | |
| --- | --- | --- | --- | --- | --- | --- | --- | --- | --- | --- | --- | --- |
|  | O | PY | IR (95% CI)  per 100 000 PY | O | PY | IR (95% CI)  per 100 000 PY | O | PY | IR (95% CI)  per 100 000 PY | O | PY | IR (95% CI)  per 100 000 PY |
| Women | 15 | 24 015.2 | 62.4 (30.8-94.0) | 16 | 32 373.3 | 49.4 (25.2-73.6) | 36 | 50 516.7 | 71.2 (47.9-94.5) | 67 | 106 905.2 | 62.6 (47.6-77.6) |
| Men | 132 | 77 081.4 | 171.2 (142.0-200.4) | 205 | 91 868.4 | 223.1 (192.5-253.6) | 259 | 128 048.2 | 202.2 (177.6-226.9) | 596 | 296 998.0 | 200.6 (184.5-216.7) |
