# Supplementary material for: Trends in the risk of myocardial infarction among HIV-1-infected individuals relative to the general population in France: Impact of gender and immune status
Source: PLoS One. 2019 Jan 16;14(1):e0210253. doi: 10.1371/journal.pone.0210253 (PMC6334967; doi:10.1371/journal.pone.0210253)
Supplement: S2 Table — Abbreviations: HCV, hepatitis C virus; CI, confidence interval; O/E, observed/expected cases; SIR, standardized incidence ratio; HCV Ab, hepatitis C antibody. P values for the SIRs were obtained by Poisson regression by comparison two by two and their 95% CIs were estimated with an exact method based on the Poisson distribution. *P values for the SIRs were obtained by Poisson regression by comparison between men and women in each calendar period. (DOCX) [file pone.0210253.s002.docx]

**S2 Table**.

| Men | Early cART era  (2000-2002) | | Intermediate cART era  (2003-2005) | | Late cART era  (2006-2009) | | *P* value  2000-2002  vs.  2003-2005 | *P* value  2006-2009  vs.  2003-2005 |
| --- | --- | --- | --- | --- | --- | --- | --- | --- |
|  | O/E | SIR (95% CI) | O/E | SIR (95% CI) | O/E | SIR (95% CI) |  |  |
| HCV Ab+ | 14/7.03 | 1.99 (1.09-3.34) | 30/9.66 | 3.10 (2.09-4.43) | 40/19.59 | 2.04 (1.46-2.78) | 0.170 | 0.083 |
| HCV Ab- | 118/90.08 | 1.31 (1.08-1.57) | 175/108.29 | 1.62 (1.39-1.87) | 219/211.22 | 1.04 (0.90-1.18) | 0.078 | <0.001 |
| *P value** |  | 0.138 |  | 0.001 |  | <0.001 |  |  |
