# Supplementary figures and images for: Trends in the risk of myocardial infarction among HIV-1-infected individuals relative to the general population in France: Impact of gender and immune status
Source: PLoS One. 2019 Jan 16;14(1):e0210253. doi: 10.1371/journal.pone.0210253 (PMC6334967; doi:10.1371/journal.pone.0210253)

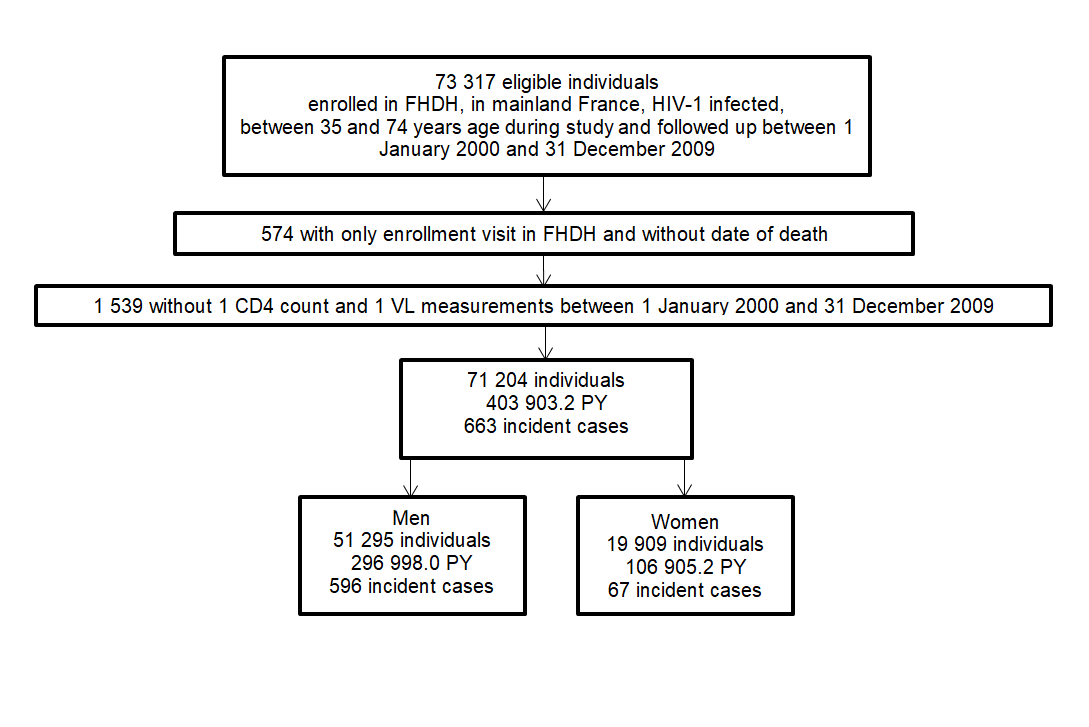

Supplement: S1 Fig — Abbreviations: FHDH, French Hospital Database on HIV; VL, viral load; MI, myocardial infarction; PY, person-years. (TIF) [file pone.0210253.s003.tif]

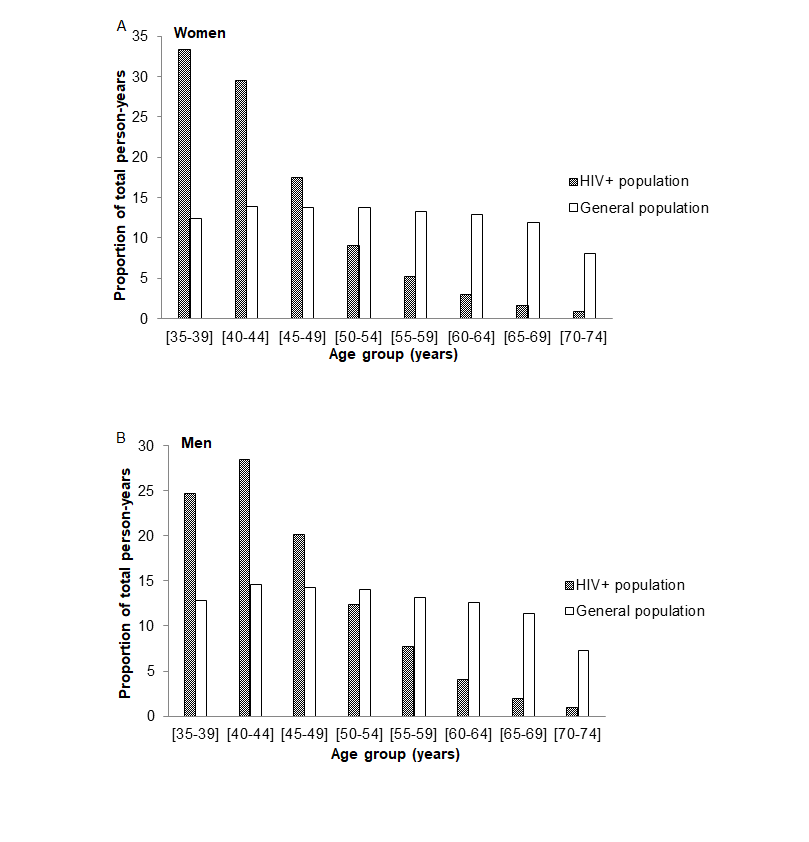

Supplement: S2 Fig — (TIF) [file pone.0210253.s004.tif]

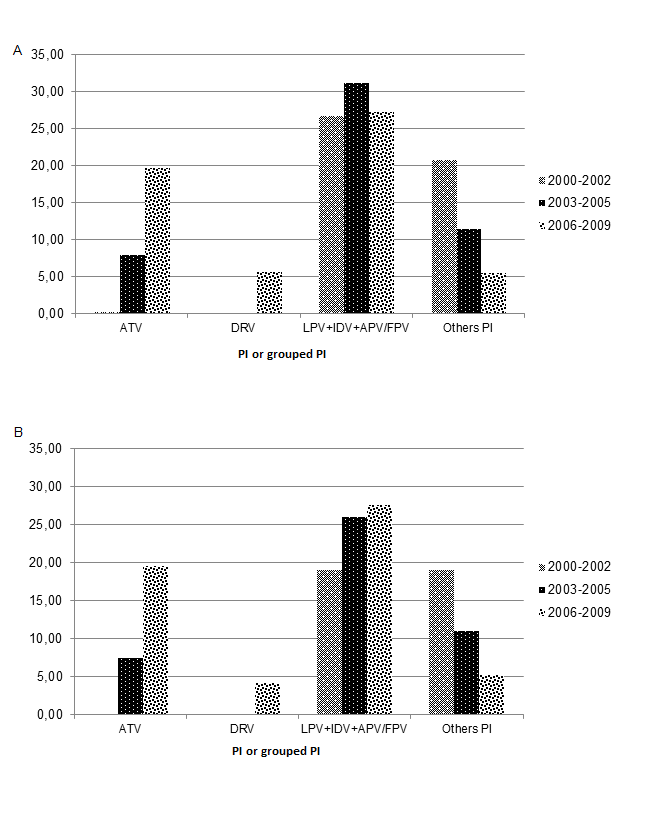

Supplement: S3 Fig — Trends in the use of protease inhibitors (PI) in men (A) and women (B) in FHDH-ANRS CO4. Abbreviations: ATV, atazanavir; DRV, darunavir; LPV, lopinavir; IDV, indinavir; APV, amprenavir; FPV, fos-amprenavir. (TIF) [file pone.0210253.s005.tif]
